# Refinement of Copper(II) Azide with 1‐Alkyl‐5*H*‐tetrazoles: Adaptable Energetic Complexes

**DOI:** 10.1002/anie.202002823

**Published:** 2020-04-21

**Authors:** Maximilian H. H. Wurzenberger, Marcus Lommel, Michael S. Gruhne, Norbert Szimhardt, Jörg Stierstorfer

**Affiliations:** ^1^ Department of Chemistry University of Munich (LMU) Butenandtstrasse 5–13 (D) 81377 München Germany

**Keywords:** azides, copper complexes, nitrogen heterocycles, phlegmatization, X-ray diffraction

## Abstract

A concept for stabilizing highly sensitive and explosive copper(II) azide with 1‐N‐substituted tetrazoles is described. It was possible to stabilize the system by the use of highly endothermic, nitrogen‐rich ligands. The sensitivities of the resulting energetic copper coordination compounds can be tuned further by variation of the alkyl chain of the ligands and by phlegmatization of the complexes with classical additives during the synthesis. It is demonstrated, using the compound based on 1‐methyl‐5*H*‐tetrazole ([Cu(N_3_)_2_(MTZ)], **1**) that this class of complexes can be applied as a potential replacement for both lead azide (LA) and lead styphnate (LS). The complex was extensively investigated according to its chemical (elemental analysis, single‐crystal and powder X‐ray diffraction, IR spectroscopy, scanning electron microscopy) and physico‐chemical properties (differential thermal analysis, sensitivities towards impact, friction, and electrostatic discharge) compared to pure copper(II) azide.

## Graphical abstract

**Tuning the boom**: Volatile copper(II) azide is stabilized by the addition of both tetrazole ligands and phlegmatizing agents. The resulting complexes can be tuned in terms of their sensitivity and serve to replace lead azide as well as lead styphnate, thus reducing the release of toxic lead into the environment.

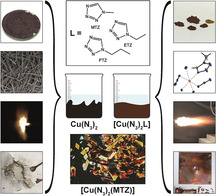

The azide anion (N_3_^−^) has attracted the attention of chemists worldwide for centuries. While some are scared because of its high nitrogen content and consequent explosive character, others are attracted for the same reason. Due to their highly versatile nature, azides are not only commonly used in organic synthesis and pharmaceutics but also in energetic materials such as in airbags, propellants, and explosives.^1^ In recent years, numerous new pentazolate (N_5_^−^) derivatives synthesized by selective C−N bond cleavage of pentazoles using mCPBA and iron glycinate generated excitement in the field.^2^ The first compound of these five‐membered heterocycles was described by Huisgen and Ugi. It was detected after a 1,3‐dipolar cycloaddition (Huisgen reaction) of a benzene diazonium chloride and lithium azide.^3^ A further current global interest is the replacement of lead‐containing explosives, particularly lead azide and lead styphnate, with safer and less toxic energetic materials.^4^ Various metal salts have been described as substitutes, mainly silver azide and rarely copper azides, although only cupric Cu(N_3_)_2_ (and not cuprous CuN_3_) might be of practical interest. However, both azides are extremely sensitive towards impact and friction. Furthermore, Cu(N_3_)_2_ is decomposed by mineral acids as well as bases and slowly forms basic cupric azides (e.g. Cu(N_3_)_2_⋅*x* Cu(OH)_2_ (*x=*1–3) and Cu(N_3_)_2_⋅8 CuO) when exposed to humidity (Figure S18 in the Supporting Information).^5^

In this work, a procedure is described for the syntheses of nitrogen‐rich copper(II) azide complexes involving 5*H*‐tetrazoles. This concept is explained using three ligands exemplarily (1‐methyl‐5*H*‐tetrazole (MTZ), 1‐ethyl‐5*H*‐tetrazole (ETZ), and 1‐propyl‐5*H*‐tetrazole (PTZ)). Moreover, many different tetrazoles, as well as triazoles, are potential candidates. The application of these compounds yielded two independent patents,^6^ which can be discussed and described scientifically. In general, it is known that metal azides (e.g. Mn, Nb, Ti, Zr, Hf, V, W, and Mo) can be stabilized with nitrogen donor ligands, although only some complexes of Cu(N_3_)_2_ have been reported in the literature and are rarely discussed as energetic materials.^7^

For the preparation of energetic coordination compounds (ECC) based on copper(II) azide, the implementation of nitrogen‐rich ligands leads to the blockage of one coordination site, resulting in stabilization in comparison to the pure metal azide (Figure 1).

**Figure 1:**
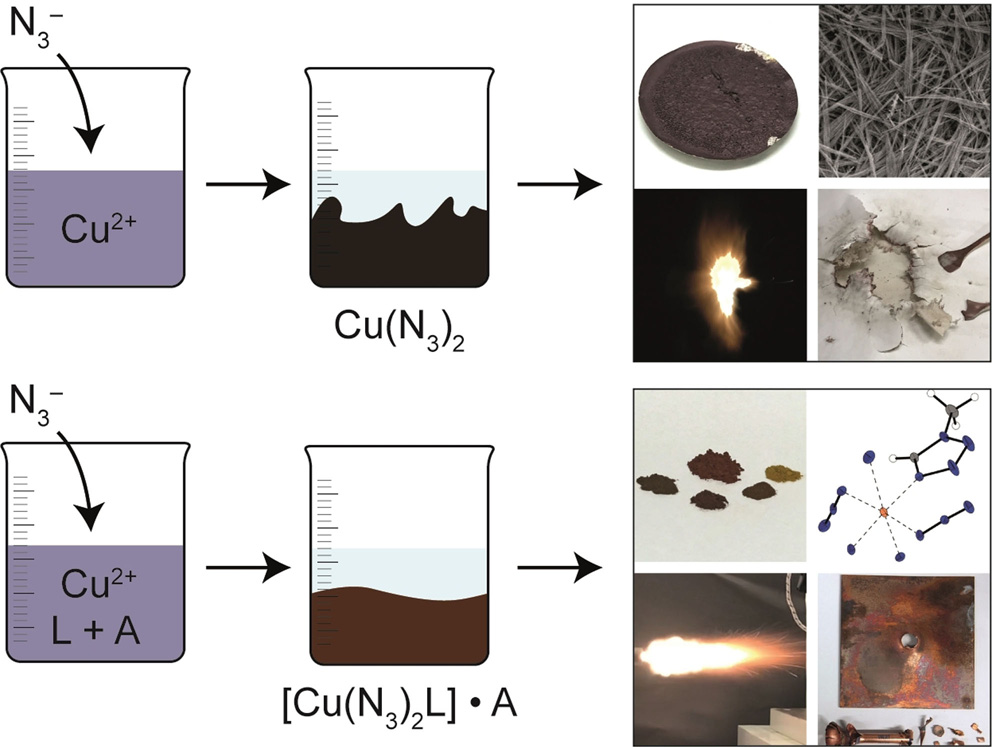
Top: Addition of azide to an aqueous solution of copper(II) leads to instant precipitation of copper azide. Safe handling is almost impossible as the material shows characteristics of a contact explosive. Bottom: Addition of azide to an aqueous solution of copper(II), ligand (L), and additive (A) leads to precipitation of a copper(II) azide complex. The resulting compound can be safely applied as primary explosive.

The azide anion is extremely toxic to the environment and all life forms, but its effect on the ecological system depends strongly on the compounds’ solubility. High water solubility implies a potent absorption through the skin and mucous membranes, leading to serious consequences for even relatively small amounts (≈10 mg).^8^ Copper(II) azide was chosen as the main building block due to the expected low solubility of the obtained compounds, resulting in a low risk to the environment. The driving force for the synthesis of the copper(II) azide complexes is the instantaneous precipitation of the compounds after the addition of sodium azide. To prevent the formation of pure copper(II) azide, an aqueous solution of sodium azide was slowly added to in situ generated complexes of soluble copper(II) salts with ligands (Scheme 1). The tetrazoles were synthesized starting from the corresponding alkyl amines or bromides.^9^ The addition of sodium azide led to the products’ formation as brown precipitates in very good yields (90–93 %). The suspensions were mechanically stirred for 10 min, filtered off, washed with water as well as ethanol, and dried in air. Single crystals suitable for X‐ray diffraction were obtained by layering concentrated aqueous and ethanolic solutions to ensure slow formation at the phase boundary. It is also possible to phlegmatize the energetic coordination compounds by using common additives such as carboxymethyl cellulose during the synthesis according to modified literature procedures.^10^

**Scheme 1:**
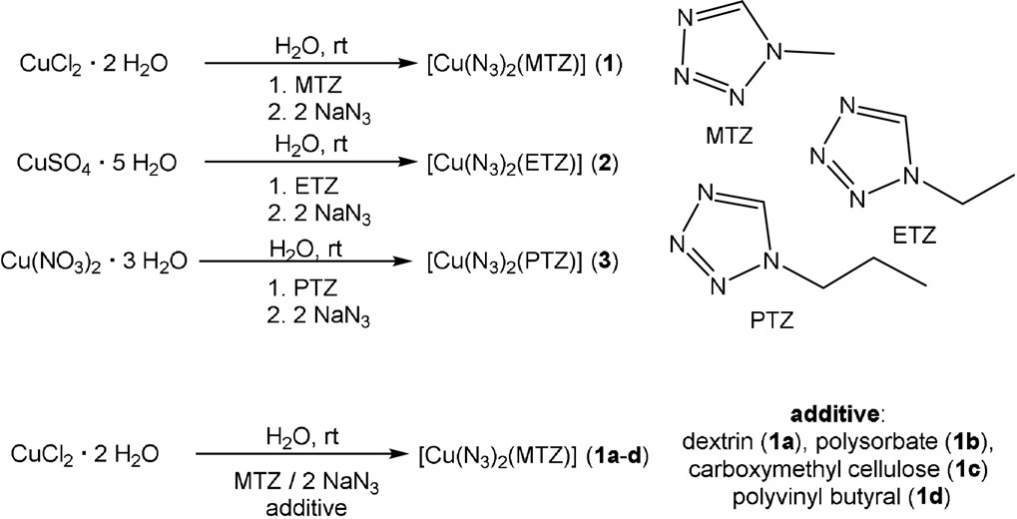
Syntheses of selected tetrazole complexes **1**–**3** as pure and as phlegmatized compounds **1 a**–**1 d**.

All compounds show a similar coordination behavior and therefore [Cu(N_3_)_2_(MTZ)] (**1**) is discussed exemplarily for all three presented complexes. The other structures can be found in the Supporting Information (Figures S3 and S4). Compound **1** crystallizes in the form of red‐brown plates in the monoclinic space group *P*2_1_/*c* with four formula units per unit cell and a calculated density of 2.036 g cm^−3^ at 123 K.^11^ The molecular unit consists of one copper(II) central cation coordinated by one MTZ ligand in equatorial position and five azido anions (Figure 2 A). The two different bridging modes of the azides (Figure 2 C) favor the formation of 2D layers (Figure 2 B). Two of three equatorial counterions bridge between the same two central metals (N4) and the other three azides link three different copper(II) atoms (N1 and N3).

**Figure 2:**
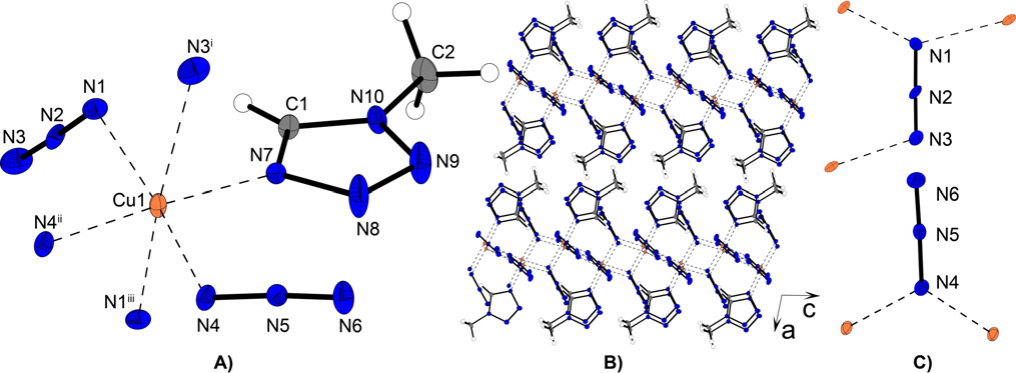
A) Copper(II) coordination environment of [Cu(N_3_)_2_(MTZ)] (**1**). Selected bond lengths (Å): Cu1−N1 2.013(3), Cu1−N3^i^ 2.563(5), Cu1−N4 2.003(3), Cu1−N7 1.996(3); selected bond angles (°): N1−Cu1−N4 171.74(12), N1−Cu1−N7 90.96(12), N1−Cu1−N3^i^ 84.99(15), N4−Cu1−N7 96.59(13). Symmetry codes: (i) −*x*, 0.5+*y*, 0.5−*z*; (ii) −*x*, 1−*y*, 1−*z*; (iii) −*x*, −0.5+*y*, 0.5−*z*. B) Polymeric structure of **1** caused by bridging anions along the *b* and *c* axes, leading to the formation of 2D layers stacked above each other along the *a* axis. C) Two different coordination modes of the azide anions in **1**.

Even though no difficulties were encountered during the synthesis or handling of the ECC, pure [Cu(N_3_)_2_(MTZ)] (**1**) must be classified as very sensitive and does not possess a no‐fire‐level against impact and friction stimuli according to standard BAM methods. Compared to pure Cu(N_3_)_2_, which shows capricious properties when handled and can even explode when slightly touched (Figure S20), compound **1** can be easily controlled.

For even safer handling and regulation of the particle size in powder form, the compound can be phlegmatized during crystallization. For the stabilization of complex **1**, the commonly used additives dextrin (**1 a**), polysorbate 80 (**1 b**), carboxymethyl cellulose (**1 c**), and polyvinyl butyral (**1 d**) were used. The phlegmatization has no significant effect on the exothermic decomposition points but in every case a more or less successful desensitization is evident. The most effective additive is carboxymethyl cellulose (**1 c**), leading to sensitivities of 2 J and 0.75 N, which are in the range of those of LA and LS (Table 1). Another approach for reducing the sensitivities of copper(II) azide complexes is the usage of tetrazole ligands with longer alkyl chains at the N1 position. The elongation of the alkyl chain to an ethyl substituent decreases the friction sensitivity to 4.5 N (**2**) and the further extension with a propyl rest to 10 N (**3**). A similar proportionality can be observed for the electrostatic discharge (ESD) values.

**Table 1:** Thermal stability and sensitivities against external stimuli of pure complexes **1**–**3** and phlegmatized **1** compared to pure cupric azide as well as commercially used lead azide (LA) and lead styphnate (LS).^12^

Cmpd.	*T*_exo_^[a]^	*IS* [J]^[b]^	*FS* [N]^[c]^	*ESD* [mJ]^[d]^
Cu(N_3_)_2_	205	≪1^5^	≪0.1	<0.28
**1**	148	<1	<0.1	0.79
**2**	134	3	4.5	33
**3**	148	2.5	10	112
**1 a**	148	<1	0.40	8.3
**1 b**	149	1.5	0.60	3.9
**1 c**	150	2	0.75	0.54
**1 d**	151	4	0.45	0.33
LA	320–360	2.5–4.0	0.1–1.0	6.0–12
LS	275–280	2.5–5.0	0.5–1.5	0.02–1.0

[a] Temperature of decomposition indicated by exothermic event according to DTA (onset temperatures at a heating rate of 5 °C min^−1^). [b] Impact sensitivity according to the BAM drop hammer (method 1 of 6). [c] Friction sensitivity according to the BAM friction tester (method 1 of 6). [d] Electrostatic discharge sensitivity (OZM XSpark10 ESD tester).

Due to the possible formation of different species, elemental analysis may represent only an average value. Confirmation of the purity of the bulk material was therefore exemplarily achieved through powder diffraction measurements of compounds **1** and **2** (Figure S5). Scanning electron microscopy (SEM) was performed to investigate the compounds’ morphology and to further examine the influence of the phlegmatization on the crystal habitus and size. It can be seen that the pure copper azide forms agglomerates made of very fine, intergrown crystalline fibers, which are the reason for its high mechanical sensitivity. Pure complex **1** shows a distribution of crystallites. The different additives used for phlegmatization make it possible to tune the crystal morphology (plate‐ or needle‐like) and corresponding size distribution (Figure 3 and Figures S6–S11).

**Figure 3:**
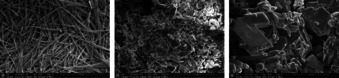
SEM images of Cu(N_3_)_2_ (8000× enlargement), pure complex **1** (6500× enlargement) and dextrinated **1 a** (6500× enlargement).

For use as a potential LA replacement, ECC **1** was tested in classical initiation capability tests. Therefore, 200 mg of the common secondary explosives pentaerythritol tetranitrate (PETN) and 1,3,5‐trinitro‐1,3,5‐triazinane (RDX) was pressed into copper shells and initiated with either pure complex **1** or the most promising phlegmatized complex (**1 c**). Further information on the test setup can be found in the Supporting Information (Figure S14). As little as 5 mg of compound **1** is able to initiate RDX reliably, making the compound an extremely efficient initiating substance (Figure 4). The initiating properties of **1** far outstrip those of recently published green primary explosives with claimed outstanding initiation efficiency.^13^ The phlegmatized complex **1 c** (50 mg) also showed positive results in initiating both PETN and RDX (Figure S15).

**Figure 4:**
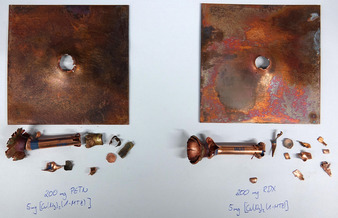
Positive PETN (left) and RDX (right) initiation tests with 5 mg of the coordination compound **1**.

Apart from its intended use as a potential replacement for lead azide, **1** can also serve as a replacement for lead styphnate for the use in priming mixtures (PM). Since the beginning of the 20th century, LS has been one of the most commonly used primary explosives in PM, which are applied to produce a flame instead of a detonation and are mostly utilized in percussion caps.^5^ Complex **1** was used for the preparation of a lead‐free PM, which was compared to a commercially available one based on lead styphnate. The PM consisted of 15 % [Cu(N_3_)_2_(MTZ)] as well as 85 % of a mixture made up of barium nitrate, aluminum, antimony trisulfide, and PETN. The created flame is sufficient for the inflammation of propellant powder (Figure 5). The resulting time–pressure curve generated with a 7.62 NATO cartridge filled with nitrocellulose powder shows an ideal gradient (Figure S17 and Table S3). The sensitivities of the new PM are similar to values of a commercial mixture (Table S2).

**Figure 5:**
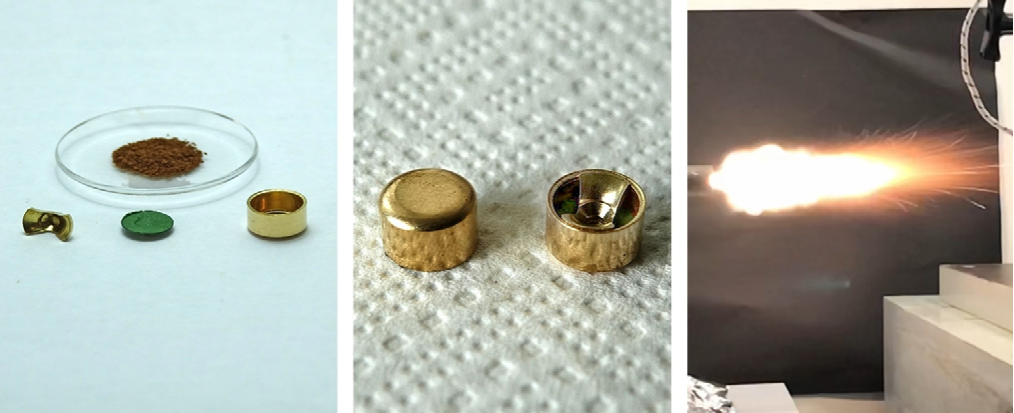
Components for the preparation of lead‐free percussion caps based on [Cu(N_3_)_2_(MTZ)] (**1**) (left and middle) and moment of ignition thereof (right).

Finally, it was successfully shown that copper(II) azide can be stabilized with *N*‐substituted tetrazole ligands and the sensitivities can be adjusted by the choice of ligand or phlegmatization during the synthesis. The resulting complexes are manageable energetic materials, which can be used as potential lead‐free primary explosives.

## Conflict of interest

The authors declare no conflict of interest.

## Supporting information

As a service to our authors and readers, this journal provides supporting information supplied by the authors. Such materials are peer reviewed and may be re‐organized for online delivery, but are not copy‐edited or typeset. Technical support issues arising from supporting information (other than missing files) should be addressed to the authors.

Supplementary
