# Supplementary material for: Refinement of Copper(II) Azide with 1‐Alkyl‐5H‐tetrazoles: Adaptable Energetic Complexes
Source: Angew Chem Int Ed Engl. 2020 Apr 21;59(30):12367–70. doi: 10.1002/anie.202002823 (PMC7383744; doi:10.1002/anie.202002823)
Supplement: Supplementary file 1 — Supplementary [file ANIE-59-12367-s001.pdf]

Angewandte Chemie
Eine Zeitschrift der Gesellschaft Deutscher Chemiker

Supporting Information

Refinement of Copper(II) Azide with 1-Alkyl-5*H*-tetrazoles: Adaptable Energetic Complexes

*Maximilian H. H. Wurzenberger, Marcus Lommel, Michael S. Gruhne, Norbert Szimhardt, and Jörg Stierstorfer**

anie_202002823_sm_miscellaneous_information.pdf

Author Contributions

J.S. Conceptualization: Equal; Project administration: Lead; Resources: Lead; Supervision: Lead; Validation: Lead; Writing - Original Draft: Supporting; Writing - Review & Editing: Lead
M.W. Writing - Original Draft: Lead
M.L. Investigation: Equal; Software: Lead; Visualization: Lead
M.G. Investigation: Equal; Software: Equal; Validation: Equal; Writing - Review & Editing: Equal
N.S. Conceptualization: Lead.

Supplementary Information

Table of Contents

1. Experimental part and general methods

2. IR spectroscopy

3. X-ray diffraction

4. Powder diffraction

5. Scanning electron microscopy

6. DTA plots

7. Initiation capability tests

8. Priming mixtures

9. Notes on the preparation of copper(II) azide

10. References

1. Experimental part and general methods

All chemicals and solvents were employed as received (Sigma-Aldrich, Fluka, Acros, ABCR). Exothermic events of the described compounds, which indicate decomposition, are given as the extrapolated onset temperatures. The samples were measured in a range of 25–300 °C at a heating rate of 5 °C min^{-1} through differential thermal analysis (DTA) with an OZM Research DTA 552-Ex instrument. Infrared spectra were measured with pure samples on a Perkin-Elmer BXII FT-IR system with a Smith DuraSampler IR II diamond ATR. Determination of the carbon, hydrogen and nitrogen contents was carried out by combustion analysis using an Elementar Vario El (nitrogen values determined are often lower than the calculated ones due to their explosive behavior). Impact sensitivity tests were carried out according to STANAG 4489[S1] with a modified instruction[S2] using a BAM (Bundesanstalt für Materialforschung) drop hammer.[S3,S4] Friction sensitivity tests were carried out according to STANAG 4487[S5] with a modified instruction[S6] using the BAM friction tester. The classification of the tested compounds results from the "UN Recommendations on the Transport of Dangerous Goods".[S7] Additionally all compounds were tested upon the sensitivity toward electrical discharge using the OZM Electric Spark XSpark10 device.[S3] The morphology of selected samples was determined by a scanning electron microscope (SEM) NanoLab G3 (Helios). The samples were carbon-coated (BAL-TEC MED 020, Bal Tec AG) to hinder electrostatic charging and to increase the conductivity.

The obtained coordination compounds were washed with cold water and ethanol when stated, dried overnight in air and used for analytics without further purification.

CAUTION! *All investigated compounds are highly energetic materials, which show increased sensitivities toward various stimuli (e.g. elevated temperatures, impact, friction or electrostatic discharge). Therefore, proper security precautions (safety glasses, face shield, earthed equipment and shoes, leather jacket, Kevlar gloves, Kevlar sleeves and ear plugs) have to be worn while synthesizing and handling the described compounds. It is recommended not to handle more than 250 mg at once.*

Procedure for the preparation of pure copper(II) azide

The pure cupric azide was prepared according to a modified procedure outlined by STRAUMANIS and CIRULIS in 1943.[S8] Diluted aqueous hydrazoic acid was prepared by ion exchange techniques, according to literature.[S9]

Copper(II) nitrate trihydrate (4.14 mmol, 1000 mg) was dissolved in water (50 mL) and stirred magnetically. An aqueous solution of sodium azide (7.70 mmol, 500 mg, 10 mL) was added dropwise. The gluey, dark brown precipitate was filtered off and washed with water. For purification, the wet azide was brought into an enclosed container of 2–3 % hydrazoic acid (50 mL) and stored for 24 h under HN_3. During filtration, the product was washed several times with ethanol and finally with diethyl ether. After drying in air, pure copper(II) azide was obtained as a brown product with a slight reddish shine. Yield: 371 mg (2.51 mmol, 61 %).

DTA (5 °C min^{-1}) onset: 205 °C (exothermic); IR (ATR, cm^{-1}): $\tilde{\nu}$ = 2123 (vs), 2089 (vs), 1302 (m), 1260 (s), 687 (m), 582 (w), 572 (m); EA (CuN_6, 147.59) calcd.: Cu 10.37, N 60.46 %; found: too sensitive for measurement; BAM drop hammer: n.d.; friction tester: < 0.10 N; ESD: < 0.29 mJ (at grain size < 100 µm).

General procedure for the preparation of copper(II) azide complexes 1–3

Stoichiometric amounts of copper(II) chloride dihydrate (**1**, 3 mmol, 511 mg), copper(II) sulfate pentahydrate (**2**, 3 mmol, 749 mg) or copper(II) nitrate trihydrate (**3**, 3 mmol, 725 mg) and the ligand (**1**, 1-methyl-5*H*-tetrazole, 3 mmol, 252 mg; **2**, 1-ethyl-5*H*-tetrazole, 3 mmol, 294 mg; **3**, 1-propyl-5*H*-tetrazole, 3 mmol, 336 mg) were stirred mechanically in 12 mL of water. Two equivalents of sodium azide, dissolved in 5 mL water, were added dropwise within 1 min and the suspension was stirred for 15 min. The precipitated complex compounds were filtered off, washed with water and ethanol and dried in air.
Single crystals growth was achieved by overlaying an aqueous solution (8 mL) of sodium azide and the ligand with an ethanolic solution (8 mL) of copper(II) chloride dihydrate, separated by a mixture (4 mL) of water/ethanol (50/50). After 7 to 14 days crystals suitable for X-ray determination were obtained.

$[Cu(N_3)_2(MTZ)]$ (1)

ECC **1** was obtained as fine brown powder. Yield: 647 mg (2.79 mmol, 93 %).
DTA (5 °C min^{-1}) onset: 157 °C (exothermic); IR (ATR, cm^{-1}): $\tilde{\nu}$ = 3366 (vw), 3326 (vw), 3121 (m), 3026 (vw), 2955 (vw), 2638 (vw), 2570 (vw), 2074 (s), 2044 (vs), 1818 (vw), 1646 (vw), 1522 (m), 1477 (vw), 1425 (w), 1346 (w), 1297 (m), 1284 (m), 1177 (m), 1107 (m), 1067 (w), 1022 (m), 1000 (m), 912 (w), 715 (w), 682 (m), 656 (m), 603 (w), 589 (w), 411 (w); EA ($C_2H_4CuN_{10}$, 231.67) calcd.: C 10.37, H 1.74, N 60.46 %; found: C 10.29, H 1.75, N 59.84 %; BAM drop hammer: < 1 J; friction tester: < 0.10 N; ESD: 0.79 mJ (at grain size < 100 µm).

[Cu(N$_3$)$_2$(ETZ)] (2)

The complex compound **2** was received in the form of a brown precipitate. Yield: 680 mg (2.77 mmol, 92 %).

DTA (5 °C min^{-1}) onset: 134 °C (exothermic); IR (ATR, cm^{-1}): $\tilde{\nu}$ = 3367 (vw), 3322 (vw), 3115 (w), 2992 (vw), 2949 (vw), 2691 (vw), 2631 (vw), 2559 (vw), 2074 (s), 2040 (vs), 1573 (vw), 1511 (m), 1438 (w), 1382 (w), 1350 (w), 1294 (m), 1279 (m), 1204 (w), 1180 (s), 1115 (m), 1099 (m), 1082 (m), 1032 (w), 1032 (w), 1010 (m), 967 (w), 907 (w), 891 (w), 803 (w), 718 (vw), 690 (w), 678 (m), 661 (w), 647 (m), 602 (m), 593 (w), 587 (w); EA ($C_3H_6CuN_{10}$, 245.70) calcd.: C 14.67, H 2.46, N 57.01%; found: C 14.38, H 2.38, N 55.90 %; BAM drop hammer: 3 J; friction tester: 4.5 N; ESD: 33 mJ (at grain size < 100 µm).

[Cu(N$_3$)$_2$(PTZ)] (3)

Complex **3** was obtained as brown precipitate. Yield: 699 mg (2.69 mmol, 90 %).

DTA (5 °C min^{-1}) onset: 148 °C (exothermic); IR (ATR, cm^{-1}): $\tilde{\nu}$ = 3124 (w), 2975 (w), 2945 (w), 2883 (w), 2092 (s), 2041 (vs), 1511 (w), 1469 (w), 1446 (w), 1375 (w), 1342 (w), 1294 (m), 1279 (m), 1180 (m), 1118 (w), 1091 (w), 1057 (vw), 1034 (vw), 1013 (w), 903 (w), 885 (w), 758 (w), 744 (w), 716 (w), 691 (w), 662 (m), 602 (w), 592 (w), 587 (w), 410 (w); EA ($C_4H_8CuN_{10}$, 259.73) calcd.: C 18.50 H 3.10 N 53.93 %; found: C 18.34 H 3.14 N 52.95 %; BAM drop hammer: 2.5 J; friction tester: 10 N; ESD: 112 mJ (at grain size < 100 µm).

Phlegmatized [Cu(N$_3$)$_2$(MTZ)] + 6 % Dextrin (1a)

The phlegmatized compound was prepared analogous to the synthesis of dextrinated lead azide.[S10] While heating to 60 °C, dextrin from potato starch (120 mg) was added to water (36 mL) under stirring. As soon as the solution became clear, sodium azide was added (17.3 mmol, 1.12 g). An aqueous solution of copper(II) chlorate dihydrate (8.63 mmol, 1.47 g) and MTZ (8.63 mmol, 0.726 g) in water (20 mL) was prepared. To this solution of metal salt and ligand, the dextrinated sodium azide solution was added dropwise over the course of 30 min while stirring and heating at 60 °C continued. After addition, the solution was allowed to cool down and the brown complex filtered, washed with ethanol and air-dried overnight. Yield: 1.72 g (6.91 mmol, 80 %).

DTA (5 °C min^{-1}) onset: 148 °C (exothermic); IR (ATR, cm^{-1}): $\tilde{\nu}$ = 3565 (vw), 3365 (vw), 3326 (w), 3120 (m), 3025 (w), 2954 (vw), 2638 (vw), 2567 (vw), 2075 (s), 2044 (vs), 1941 (m),

1816 (w), 1521 (m), 1424 (w), 1346 (w), 1297 (m), 1282 (m), 1176 (m), 1106 (m), 1066 (w), 1021 (m), 999 (m), 912 (w), 715 (w), 681 (m), 656 (m), 603 (w), 589 (m), 471 (vw), 412 (w); BAM drop hammer: < 1 J; friction tester: 0.40 N; ESD: 8.3 mJ (at grain size < 100 µm).

Phlegmatized [Cu(N_3)$_2$(MTZ)] + 5 % Span 80 (1b)

An emulsion of Span 80 (50 mg) in water (9.8 mL) was prepared and heated to 60 °C while stirring vigorously. Copper(II) chloride dihydrate (4.32 mmol, 736 mg) and MTZ (4.32 mmol, 363 mg) were added. A solution of sodium azide (8.63 mmol, 561 mg) in water (18 mL) was added dropwise to the emulsion containing metal salt and ligand over the course of 10 min. The precipitated brown complex was filtered, washed with ethanol and air-dried overnight. Yield: 763 mg (3.08 mmol, 71 %).

DTA (5 °C min^{-1}) onset: 149 °C (exothermic); IR (ATR, cm^{-1}): $\tilde{\nu}$ = 3583 (w), 3444 (vw), 3367 (w), 3326 (w), 3118 (m), 3025 (w), 2925 (w), 2855 (w), 2637 (vw), 2571 (w), 2149 (w), 2075 (s), 2044 (vs), 1809 (w), 1739 (m), 1626 (w), 1521 (m), 1468 (w), 1424 (w), 1377 (w), 1347 (w), 1283 (m), 1272 (m), 1272 (m), 1175 (m), 1105 (s), 1066 (w), 1021 (m), 998 (m), 909 (m), 715 (w), 681 (s), 655 (s), 603 (m), 588 (m), 572(w), 474(vw), 411(w); BAM drop hammer: 1.5 J; friction tester: 0.60 N; ESD: 63 mJ (at grain size < 100 µm).

Phlegmatized [Cu(N_3)$_2$(MTZ)] + 5 % CMC (1c)

The phlegmatized compound was prepared analogous to a modified procedure, describing the production of RD1333 lead azide.[S10] Sodium carboxymethylcellulose (25 mg) with medium viscosity (400-800 cP, 2 % in H_2O at 25 °C) was dissolved in water while stirring at room temperature. Copper(II) chloride dihydrate (2.16 mmol, 368 mg) and MTZ (2.16 mmol, 182 mg) were dissolved in water (10 mL). A solution of sodium azide (4.32 mmol, 281 mg) in water (10 mL) was prepared. The solution containing copper(II) salt and ligand, as well as the solution containing the sodium azide were simultaneously added dropwise to the CMC solution. After complete addition and precipitation of the complex, it was allowed to stir for another 5 min. The brown powder was filtrated, washed with ethanol and air-dried overnight. Yield: 452 mg (1.84 mmol, 85 %).

DTA (5 °C min^{-1}) onset: 150 °C (exothermic); IR (ATR, cm^{-1}): $\tilde{\nu}$ = 3366 (vw), 3326 (vw), 3120 (w), 3025 (vw), 2953 (vw), 2637 (vw), 2565 (vw), 2544 (vw), 2074 (s), 2045 (vs), 1818 (w), 1672 (w), 1612 (w), 1522 (m), 1477 (w), 1425 (w), 1346 (w), 1283 (m), 1177 (m), 1106

(m), 1067 (w), 1022 (m), 999 (m), 912 (w), 715 (w), 682 (m), 656 (m), 603 (w), 589 (w), 413 (w); BAM drop hammer: 2 J; friction tester: 0.75 N; ESD: 3.9 mJ (at grain size < 100 µm).

Phlegmatized [Cu(N_3)$_2$(MTZ)] + 5 % PVB (1d)

A solution of polyvinyl butyral (25 mg) in methanol was prepared while stirring at room temperature. After the additive was completely dissolved, copper(II) nitrate trihydrate (2.16 mmol, 522 mg) and MTZ (2.16 mmol, 182 mg) were added and dissolved in the PVA solution. An aqueous solution of sodium azide (4.32 mmol, 281 mg) was dripped to the in situ formed nitrate complex. The precipitated complex was allowed to stir for five more minutes and, after filtration, washing with ethanol and air-drying overnight, obtained as brown powder. Yield: 508 mg (2.06 mmol, 95 %).

DTA (5 °C min^{-1}) onset: 151 °C (exothermic); IR (ATR, cm^{-1}): $\tilde{\nu}$ = 3368 (vw), 3120 (w), 3009 (vw), 2968 (vw), 2638 (vw), 2580 (vw), 2084 (vs), 2048 (vs), 1783 (vw), 1515 (m), 1483 (w), 1343 (w), 1290 (s), 1184 (m), 1105 (m), 1063 (w), 1032 (w), 1000 (m), 893 (m), 835 (vw), 812 (vw), 719 (vw), 686 (m), 655 (s), 598 (w), 587 (w), 499 (vw), 408 (w); BAM drop hammer: 4 J; friction tester: 0.45 N; ESD: 0.54 mJ (at grain size < 100 µm).

2. IR spectroscopy

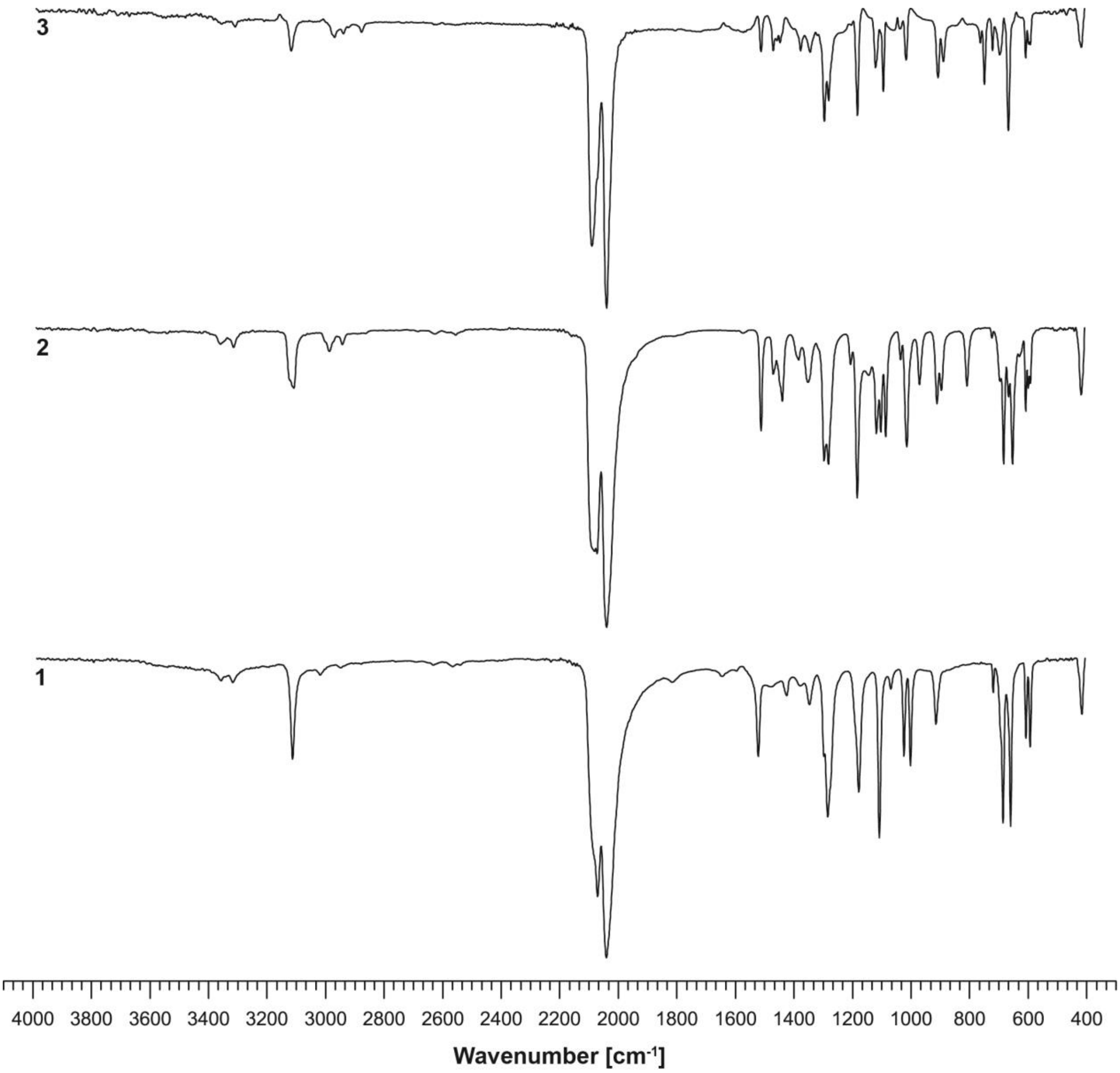

Figure S1 Infrared spectra of ECC **1**–**3**.

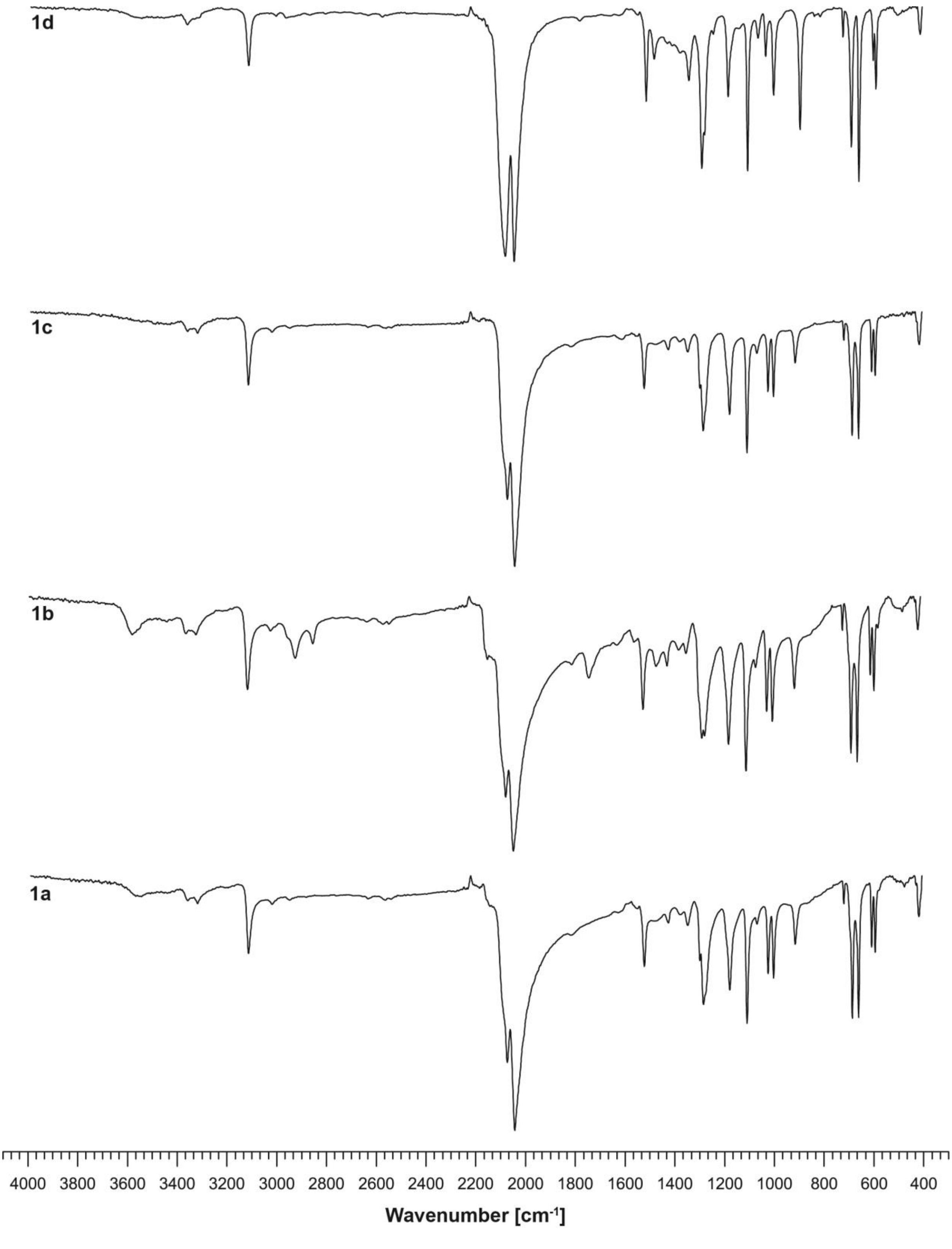

Figure S2 Infrared spectra of phlegmatized complexes **1a**–**d**.

3. X-ray diffraction

For all crystalline compounds, an Oxford Xcalibur3 diffractometer with a CCD area detector or Bruker D8 Venture TXS diffractometer equipped with a multilayer monochromator, a Photon 2 detector and a rotating-anode generator were employed for data collection using Mo-*K*α radiation (λ = 0.7107 Å). On the Oxford device, data collection and reduction were carried out using the CRYSALISPRO software[S11]. On the Bruker diffractometer, the data were collected with the Bruker Instrument Service v3.0.21, the data reduction was performed using the SAINT V8.18C software (Bruker AXS Inc., 2011). The structures were solved by direct methods (SIR-92[S12], SIR-97[S13] or SHELXS-97[S14]) and refined by full-matrix least-squares on *F*2 (SHELXL[S14]) and finally checked using the PLATON software[S15] integrated in the WinGX[S16] software suite. The non-hydrogen atoms were refined anisotropically and the hydrogen atoms were located and freely refined. The absorptions were corrected by a SCALE3 ABSPACK or SADABS Bruker APEX3 multiscan method[S17,S18]. All DIAMOND2 plots are shown with thermal ellipsoids at the 50 % probability level and hydrogen atoms are shown as small spheres of arbitrary radius.

Table S1. Crystallographic data of **1–3**.

	**1**	**2**	**3**
Formula	$C_2H_4CuN_{10}$	$C_6H_{12}Cu_2N_{20}$	$C_8H_{16}Cu_2N_{20}$
FW [g mol^{-1}]	231.69	491.44	519.49
Crystal system	monoclinic	triclinic	triclinic
Space Group	$P2_1/c$	$P{-}1$	$P{-}1$
Color / Habit	red-brown plate	yellow platelet	brown block
Size [mm]	0.05 x 0.16 x 0.21	0.01 x 0.03 x 0.04	0.03 x 0.05 x 0.10
a [Å]	11.4746(7)	6.2573(3)	6.3931(5)
b [Å]	6.1867(3)	10.7256(4)	10.6917(8)
c [Å]	10.8165(5)	12.8661(5)	14.0603(11)
α [°]	90	98.438(2)	107.276(2)
β [°]	100.078(5)	98.221(2)	90.120(2)
γ [°]	90	90.230(1)	90.221(2)
V [Å^3]	756.01(7)	845.09(6)	917.70(12)
Z	4	2	2
$\rho_{calc.}$ [g cm^{-3}]	2.036	1.931	1.880
μ [mm^{-1}]	2.858	2.563	2.365
F(000)	460	492	524
$\lambda_{MoK\alpha}$ [Å]	0.71073	0.71073	0.71073
T [K]	123	103	102
θ Min–Max [°]	4.4, 26.0	2.3, 26.0	2.8, 26.4
Dataset	-14: 13; -3: 7; -9: 13	-7: 7; -13: 13; -15: 15	-7: 7; -13: 13; -17: 17
Reflections collected	2684	8592	12240
Independent refl.	1547	3320	3702
R_{int}	0.032	0.029	0.031
Observed reflections	1326	2702	3439
Parameters	119	255	273
R_1 (obs)[a]	0.0406	0.0366	0.0722
wR_2 (all data)[b]	0. 1069	0.0926	0.1837
GooF[c]	1.07	1.07	1.18
Resd. Dens. [e Å^{-3}]	-0.90, 1.02	-0.51, 1.89	-0.99, 2.16
Absorption correction	multi-scan	multi-scan	multi-scan
CCDC	1984071	1984070	1984069

a) $R_1 = \Sigma||F_0|- |F_c||/ \Sigma|F_0|$; b) $wR_2 = [\Sigma[w(F_0^2-F_c^2)^2]/\Sigma[w(F_0)^2]]^{1/2}$; $w = [\sigma c^2(F_0^2)+(xP)^2+yP]^{-1}$ and $P=(F_0^2+2F_c^2)/3$; c) GooF = $\{\Sigma[w(F_o^2-F_c^2)^2]/(n-p)\}^{1/2}$ (n = number of reflections; p = total number of parameters).

Copper(II) azide complex **2** crystallizes as yellow platelets in the triclinic space group *P*−1 with two formula units per unit cell and a calculated density of 1.931 g cm^{-3} at 103 K. The molecular unit is built of two different copper(II) cations, each coordinated octahedrally by one ligand in equatorial position and five bridging azide anions (Figure S3). The coordination spheres show strong Jahn-Teller distortions along the N14–Cu1–N12^{i} and N9–Cu2–N11^{iv} axes and the azido ligands possess the same bridging behavior like in compound **1** again forming 2D-polymeric layers.

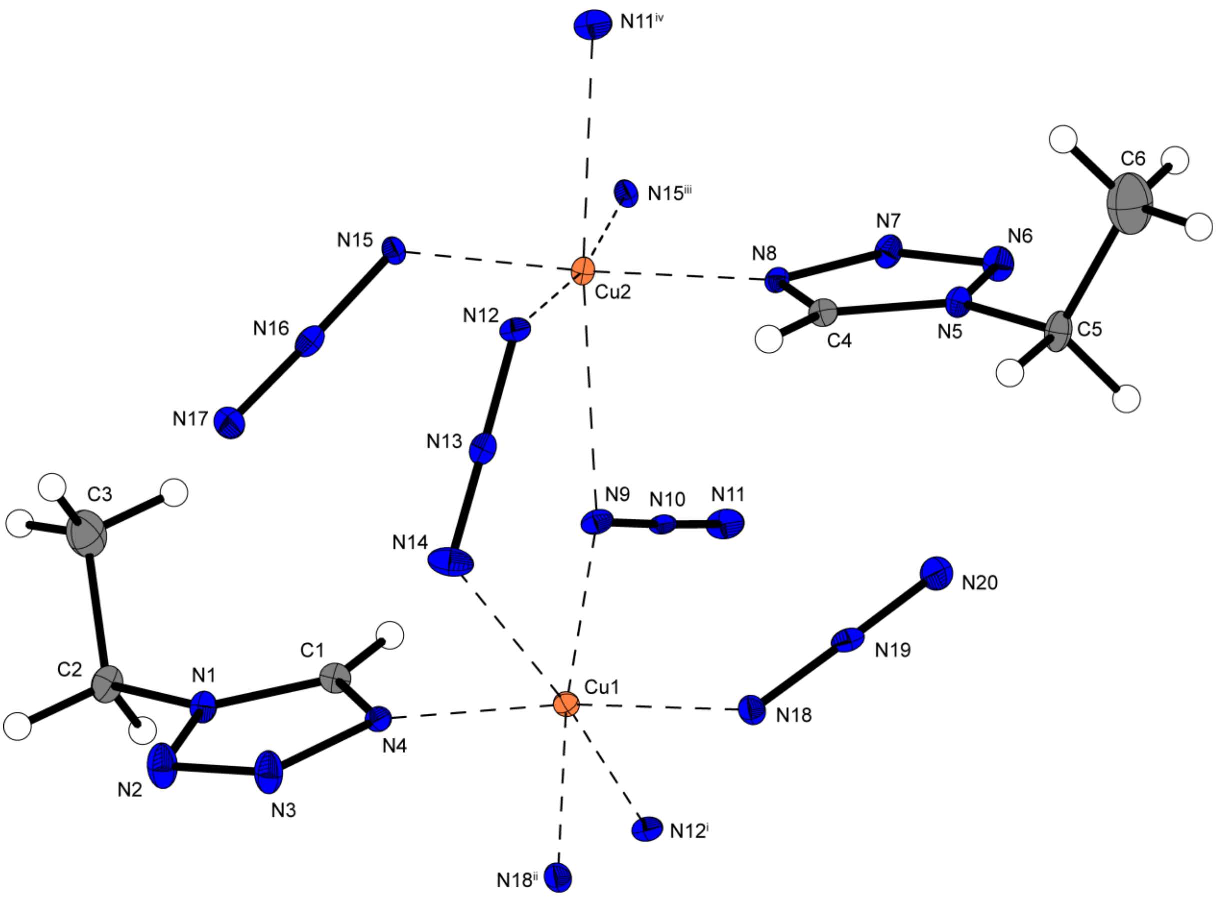

Figure S3. Coordination environment of [Cu(N_3)$_2$(ETZ)] (**2**). Selected bond lengths (Å): Cu1–N4 1.996(3), Cu1–N9 2.001(3), Cu1–N14 2.594(3), Cu1–N18^{ii} 2.010(3), Cu2–N8 1.993(3), Cu2–N9 2.545(3), Cu2–N11^{iv} 2.600(3), Cu2–N12 2.008(3); selected bond angles (°): N4–Cu1–N9 91.87(14), N4–Cu1–N14 85.33(12), N9–Cu1–N14 85.11(12), N8–Cu2–N9 89.54(12), N8–Cu2–N12 91.51(12), N9–Cu2–N12 89.83(12). Symmetry codes: (i) −1+x, y, z; (ii) 2−x, 1−y, −z; (iii) 1−x, 2−y, −z; (iv) −1+x, y, z.

ECC **3** crystallizes as brown blocks in the triclinic space group *P*−1. It possesses two formula units per unit cell and the lowest calculated density (1.880 g cm^{-3} at 102 K) of all three compounds. Again, the molecular unit consists of two different metal(II) cations, each coordinated octahedrally by one ligand in equatorial position and five bridging azide anions (Figure S4). The coordination spheres show strong Jahn-Teller distortions along the N15^{iii}–Cu1–N17 and N8–Cu2–N10^{ii} axes and the azido ligands possess the same bridging behavior like in compound **1** and **2** leading to the formation of layers.

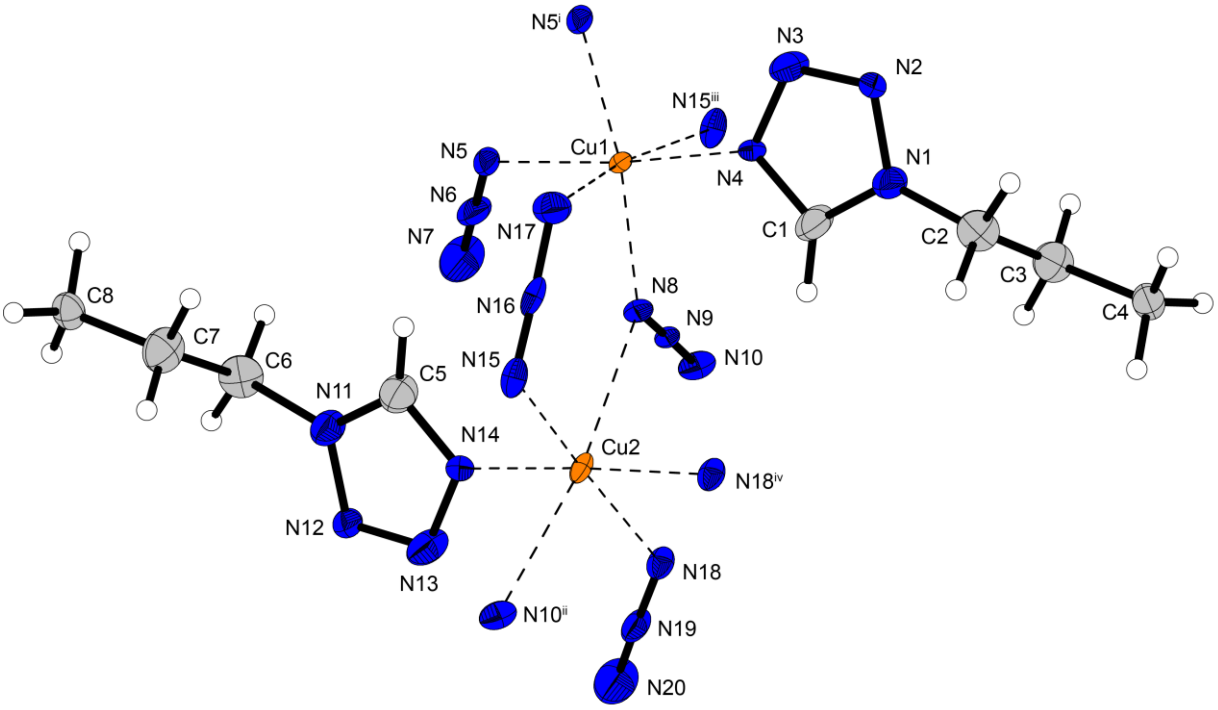

Figure S4. Coordination environment of $[Cu(N_3)_2(PTZ)]$ (**3**). Selected bond lengths (Å): Cu1–N4 2.000(6), Cu1–N5 2.006(7), Cu1–N8 2.005(7), Cu1–N5^{i} 2.000(7), Cu1–N15^{iii} 2.592(7), Cu1–N17 2.639(7), Cu2–N8 2.637(7), Cu2–N14 1.989(6), Cu2–N15 2.016(7), Cu2–N18 1.994(7), Cu2–N10^{ii} 2.593(7); selected bond angles (°): N5–Cu1–N4 95.9(2), N5–Cu1–N5 79.0(3), N4–Cu1–N5 174.8(2), N8–Cu1–N5 93.6(3), N14–Cu2–N8 96.0(3), N14–Cu2–N18 174.1(2), N8–Cu2–N18 78.3(3), N14–Cu2–N15 91.5(3), N18–Cu2–N15 171.9(3), N8–Cu2–N15 94.2(2), N8–Cu2–N10 171.3(2). Symmetry codes: (i) 1−x, 1−y, 1−z; (ii) 1+x, y, z; (iii) −1+x, y, z; (iv) 2−x, −y, 1−z.

4. Powder diffraction

X-ray powder experiments were performed on a Gunier diffractometer (Huber G644) with Mo-Kα1 radiation (λ = 0.7093 Å, quartz monochromator) in Lindemann capillaries (0.7 mm diameter). The angle calibration was performed with electronic grade germanium. In the 2θ range between 4 and 34° with an increment of 0.04°, 750 data points were collected with a counting rate of 10 s for each increment. The Rietveld parameters were analyzed with the program FullProf.[S19]

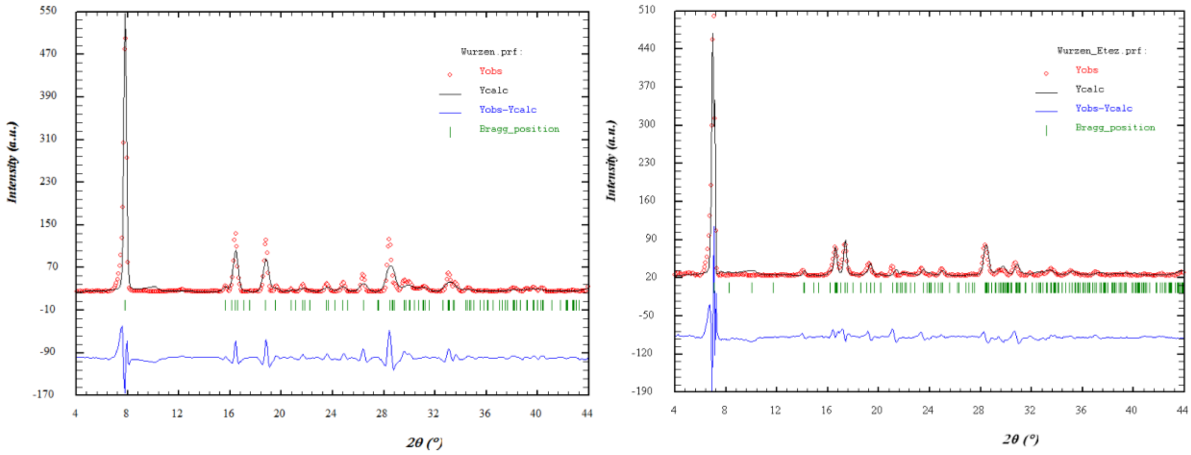

[Cu(N_3)$_2$(MTZ)] (**1**) [Cu(N_3)$_2$(ETZ)] (**2**)

Figure S5. Powder diffraction of compounds 1 and 2. Small aberrations of observed and calculated data are caused by the temperature difference of single crystal and powder diffraction experiments.

5. Scanning electron microscopy

Since all of the prepared compounds differ in their physicochemical properties and appearance, scanning electron microscopy (SEM) was performed to investigate the morphology of pure copper azide as well as the prepared coordination compound with (**1a**–**1d**) and without (**1**) additives.

The pure azide consists of thin fibers with a diameter of less than 1 µm, forming intergrown agglomerates (Figure S6). This is also represented in the macroscopic scale, as the dry compound does not form a fine powder but keeps the shape of the filter paper and crumbles into large chunks.

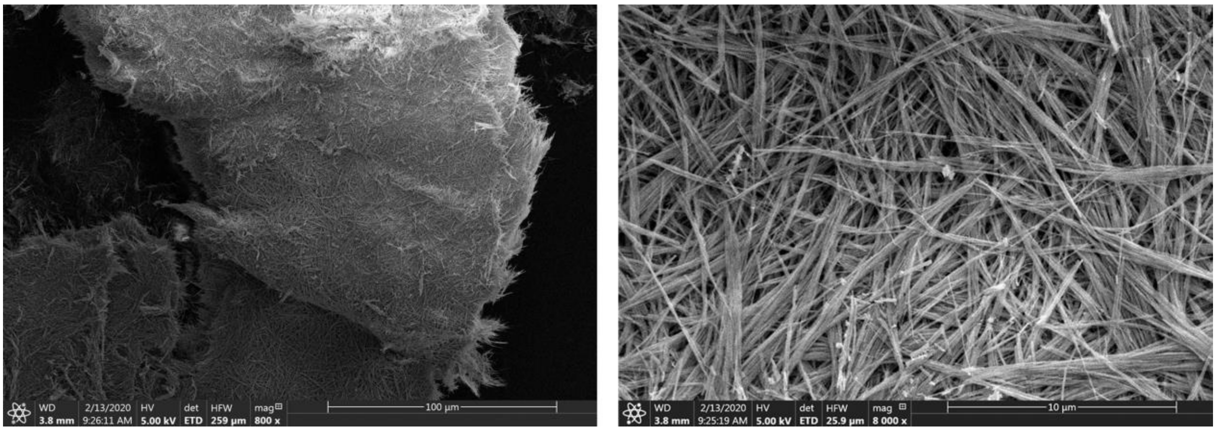

Figure S6. SEM images with 800x magnitude (left) and 8000x magnitude (right) of pure $Cu(N_3)_2$.

The coordination compound (**1**) shows a completely different crystal habit, as small crystallites are homogenously distributed (Figure S7). Most of them have a plate-like morphology, rounded edges and a size in the range of 0.5–5 µm. This can also be confirmed by the compound's macroscopic appearance, as it precipitates as a fine powder and retains this morphology after drying.

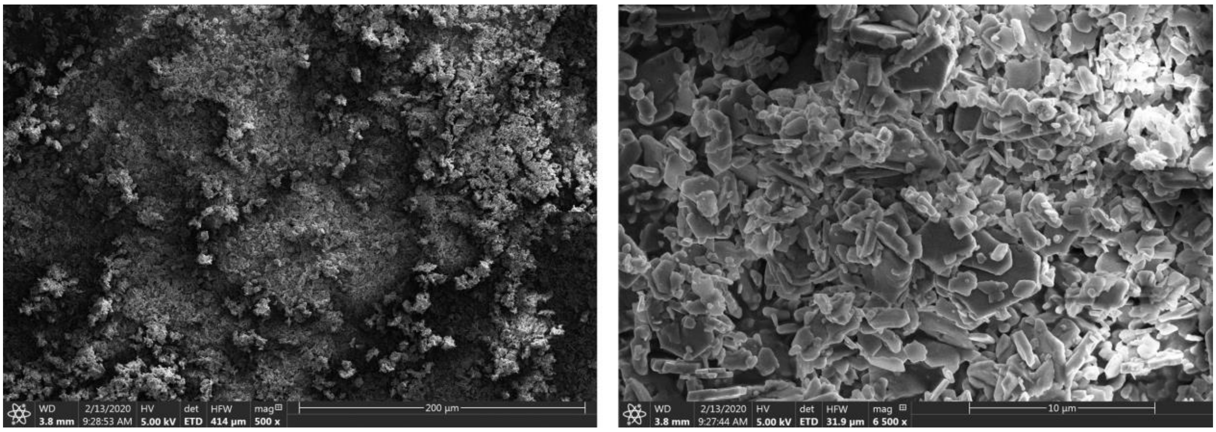

Figure S7. SEM images with 500x magnitude (left) and 6500x magnitude (right) of compound **1**.

The dextrinated complex (**1a**) shows a similar overall morphology but with bigger crystallites (up to 30 µm) tending to take on more geometric shapes (Figure S8).

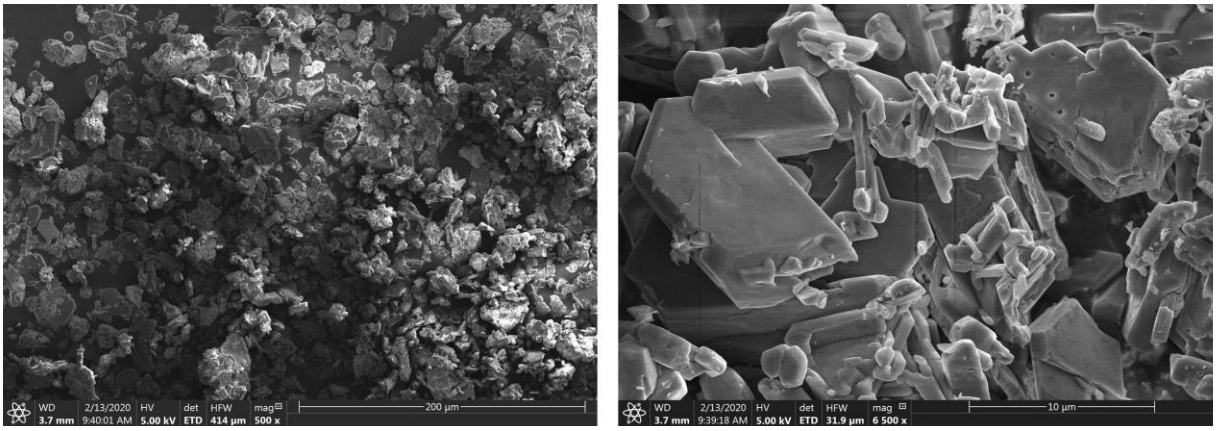

Figure S8. SEM images with 500x magnitude (left) and 6500x magnitude (right) of compound **1a**.

The complex **1b** precipitated from an aqueous solution of polysorbate (Span 80) shows a larger particle size distribution and forms even bigger chunks (up to 80 µm) which are partially intergrown and possess soft edges (Figure S9).

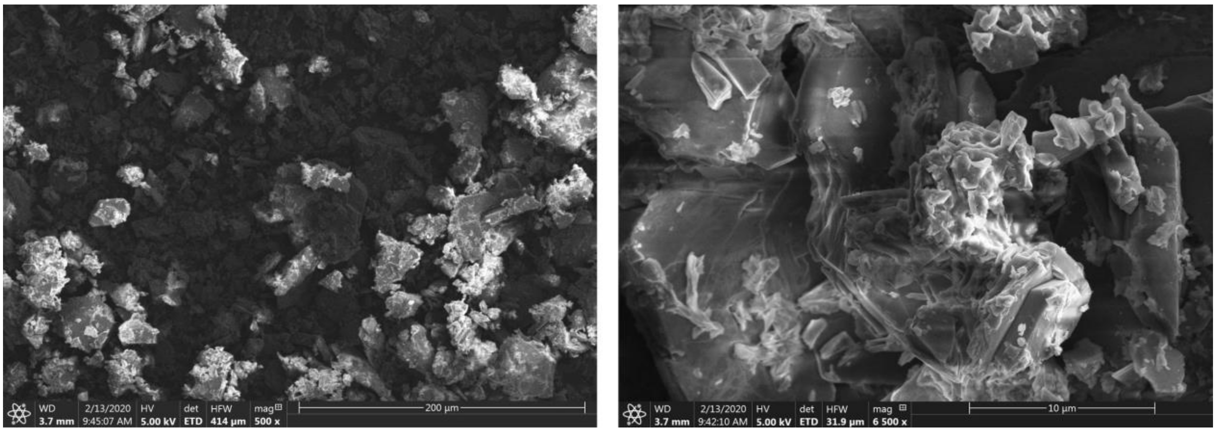

Figure S9. SEM images with 500x magnitude (left) and 6500x magnitude (right) of compound **1b**.

Interestingly, compound **1c**, which is prepared using carboxymethyl cellulose as an additive, forms agglomerates consisting of two different crystal morphologies (Figure S10). Firstly, a plate-like structure (as present in pure complex **1**) can be found, as well as a needle-like species. With no structures being much bigger than 10 µm, the overall morphology seems to be very compact due to space-filling needles between layers of platelets.

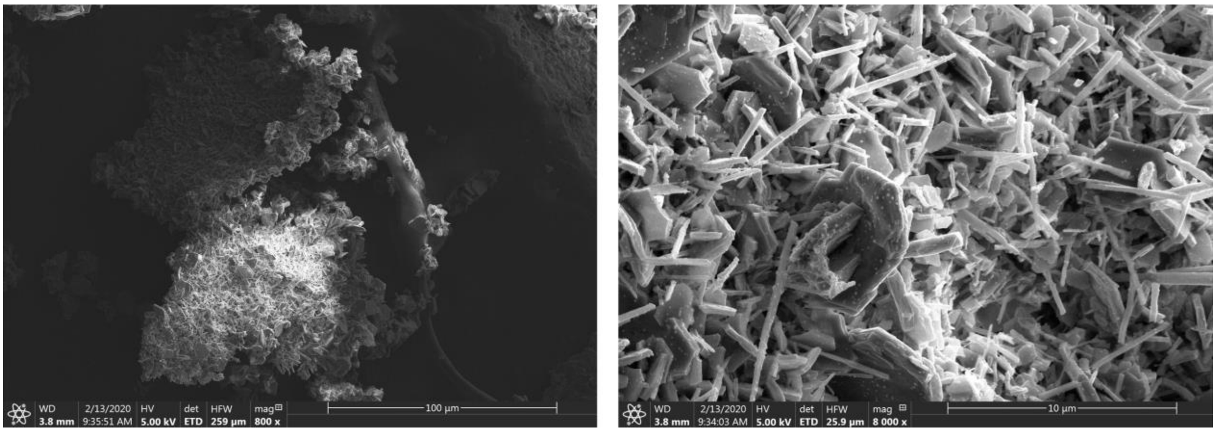

Figure S10. SEM images with 800x magnitude (left) and 8000x magnitude (right) of compound **1c**.

The complex **1d**, precipitated out an aqueous solution of polyvinyl butyral, shows a morphology with the smallest structures (Figure S11) in this row. With very short needle-like crystals in the range of 0.2–2 µm, the formation of agglomerates is facilitated as well as a homogeneous general morphology.

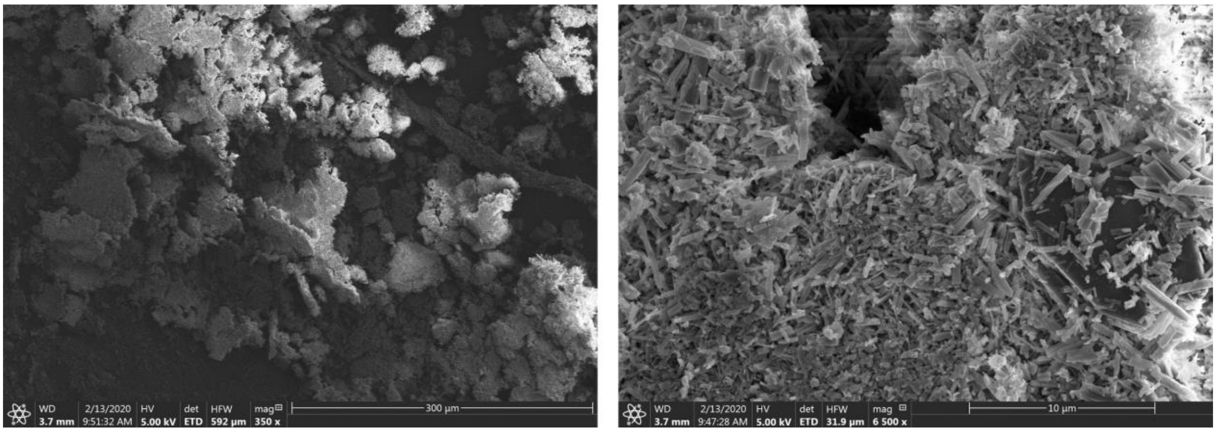

Figure S11. SEM images with 350x magnitude (left) and 6500x magnitude (right) of compound **1c**.

6. DTA plots

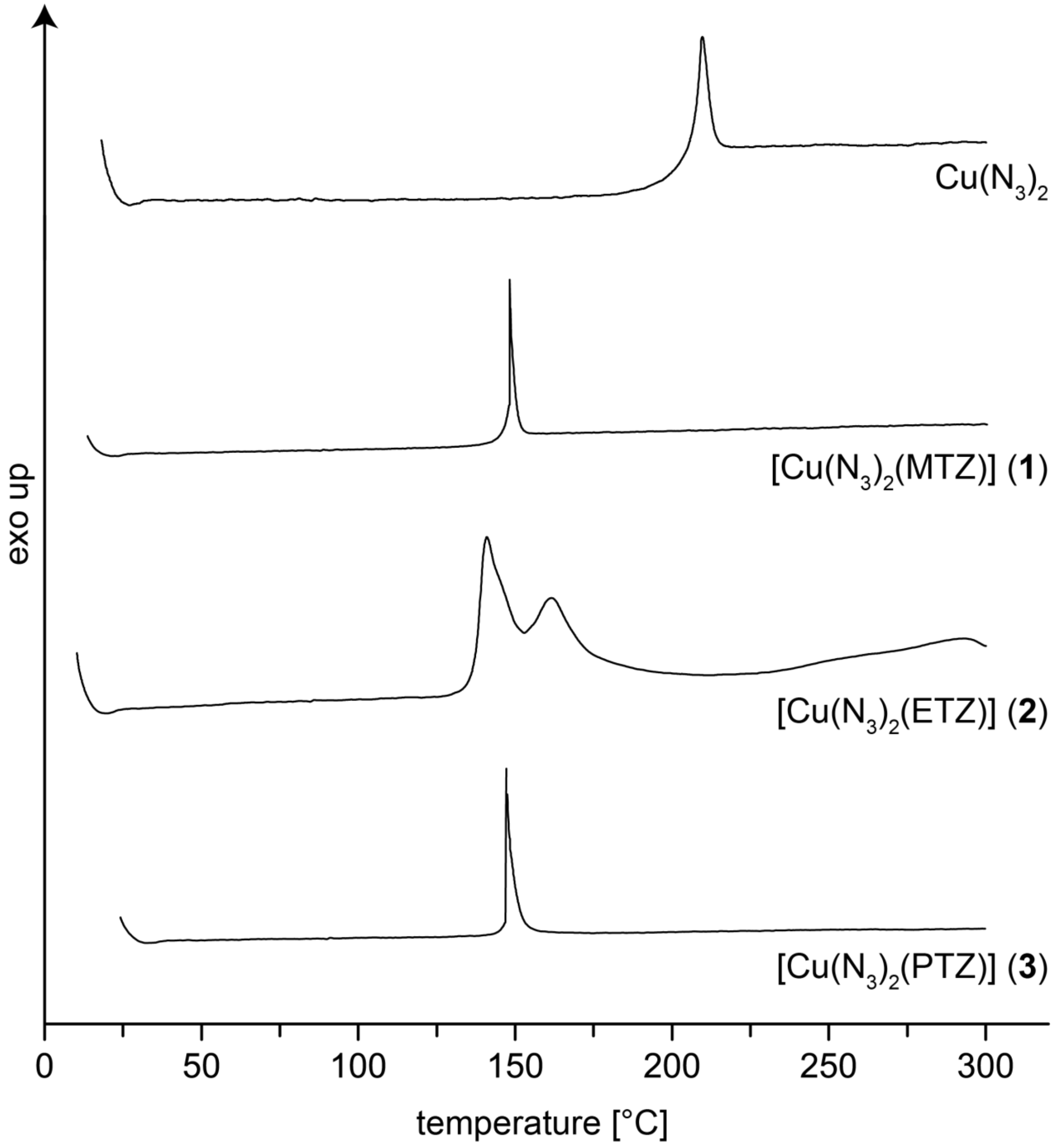

Figure S12. DTA plots of copper(II) azide and ECC **1**–**3**.

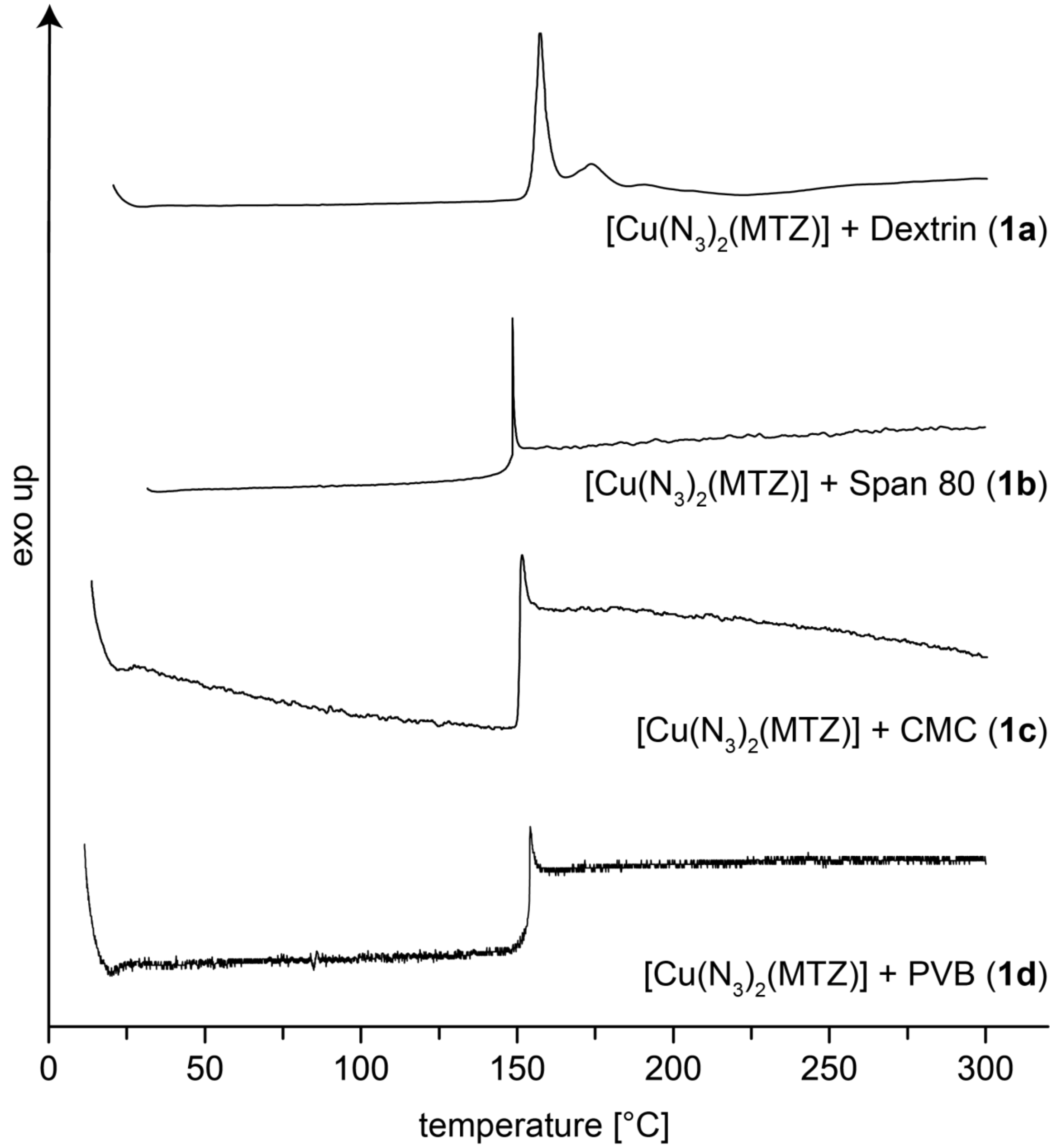

Figure S13. DTA plots of phlegmatized complexes **1a**–**c**.

7. Initiation capability tests

As the initiating capability of a compound indicates its suitability as a primary explosive, selected ECC were tested in initiation experiments. The compound to be evaluated was loosely filled on top of a pressed (8 kg weight) main charge (200 mg of PETN or RDX) in a copper shell (Figure S14). The primary explosive was ignited using an electrical ignitor.

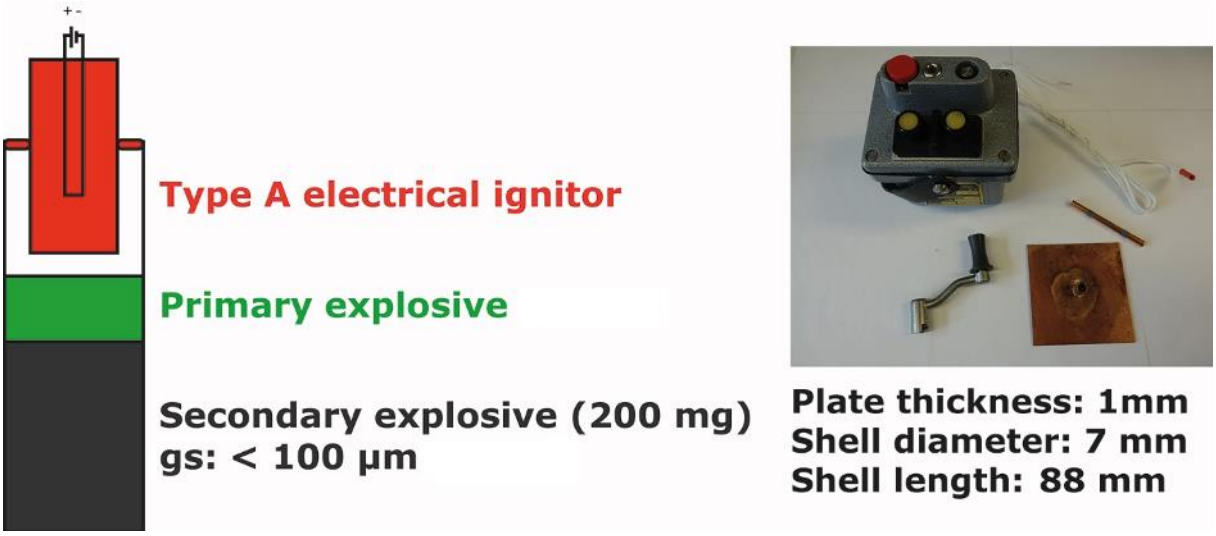

Figure S14. Schematic test setup (left) and used equipment (right) for the initiation capability tests.

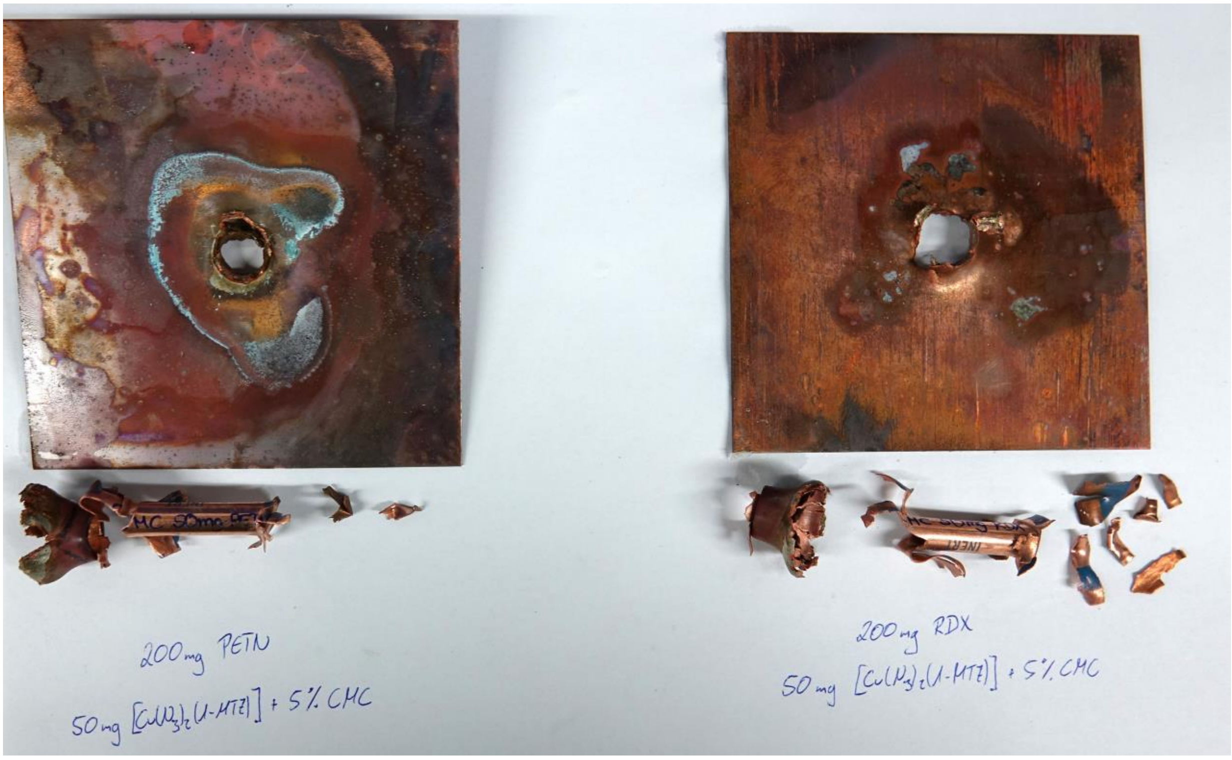

Figure S15. Positive PETN (left) and RDX (right) initiation tests with 50 mg of the phlegmatized coordination compound **1c**.

8. Priming mixtures

In order to test the suitability in priming mixtures (PM), [Cu(N_3)$_2$(MTZ)] (**1**) was applied as a lead styphnate (LS) replacement in a priming composition similar to the so called FA-956.[S20] Instead of using 41 % of primary explosive by mass (37 % LS, 4 % tetrazene), the mixture was tested with 15 % of [Cu(N_3)$_2$(MTZ)] as a primary explosive. For obtaining homogenous priming mixtures in lab scale, weighted quantities of all compounds were brought into a sample container and placed into a Heidolph Reax 2 overhead shaker. After several hours at 60 rpm, a homogenous mixture was attained, which was further characterized regarding sensitivities, thermal behavior, the produced flame. Therefore, the PM was filled in commonly used large-rifle percussion primer consisting of a brass primer cup (B) covered with a paper disc (C), which is pressed onto the mixture. Lastly an anvil (D) is pressed on top of the paper (Figure S16). As soon as the firing pin hits the primer cup (A), mechanical stimulus ignites the priming mixture (B), which is confined by the anvil (D) and subjected to impact and friction.

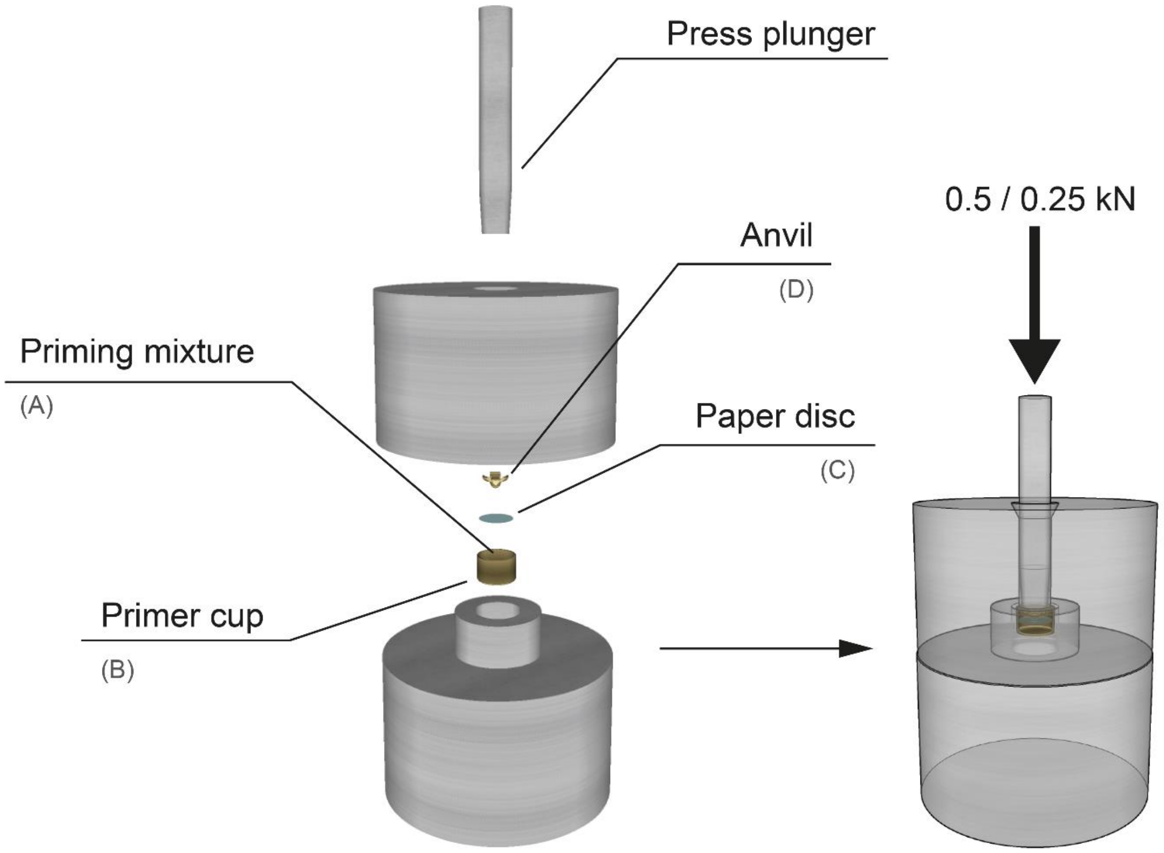

Figure S16. Primer processing.

Table S2. Sensitivities of the new PM compared to a commercially available one

Compound	*IS* [J][a]	*FS* [N] [b]	*ESD* [mJ][c]
Lead-free mixture	1	10	3.3
Commercial mixture	1	30	0.79

[a] Impact sensitivity according to the BAM drop hammer (method 1 of 6). [b] Friction sensitivity according to the BAM friction tester (method 1 of 6). [c] Electrostatic discharge sensitivity (OZM ESD tester).

A primer filled with the new mixture was pressed into a 7.62 mm cartridge and the priming mixture ignited with the impact of a firing pin. Nitrocellulose was used as propellant. The building up gas pressure was measured and compared to a cartridge with a commercial large rifle primer. The results can be found in Table S3 and Figure S17. The generated gas pressure [bar] is plotted against time [ms].

Table S3. Results of the gas pressure measurements.

Compound	Pressure [bar]	Velocity [m/s]	Energy [J]
Lead-free mixture	2998.0	822.1	3228
Commercial mixture	3232.4	839.8	3366

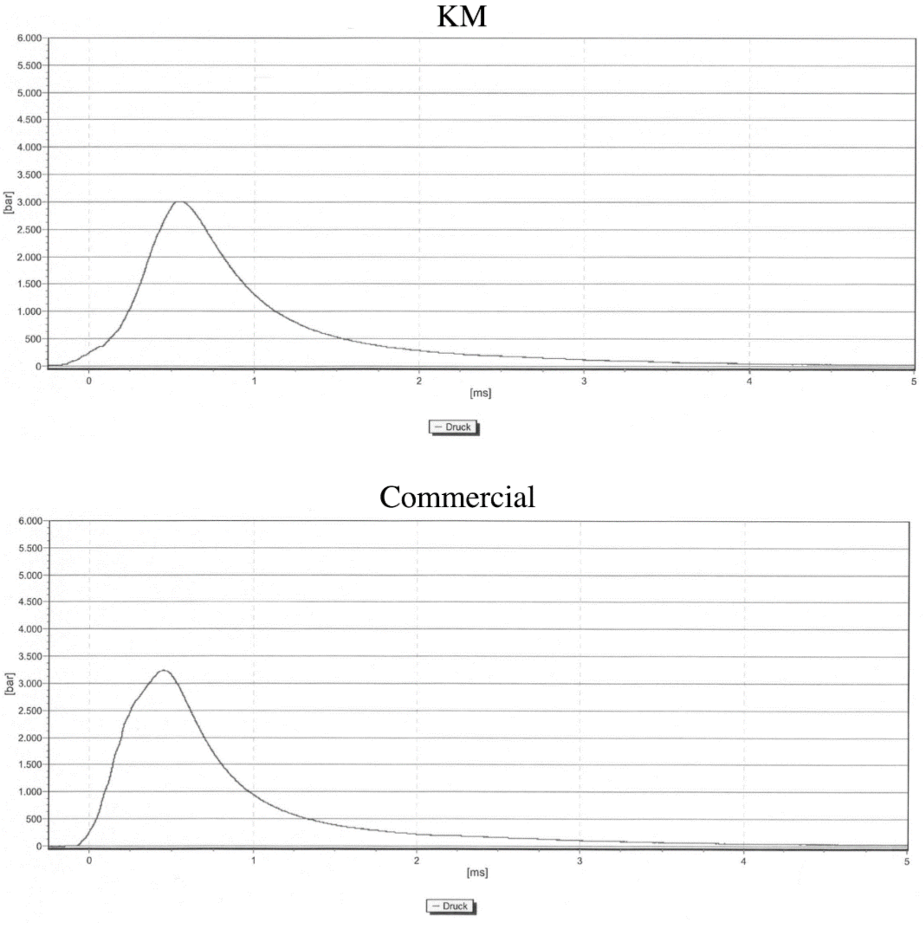

Figure S17. Generated gas pressure of two mixtures, plotted against time.

9. Notes on the preparation of copper(II) azide

CAUTION! *The pure copper azide is very sensitive toward all external stimuli and shows characteristics of a contact explosive!*

Cupric azide was prepared as outlined in the Experimental Section, according to STRAUMANIS and CIRULIS.[S8] After the addition of azide to a solution of copper nitrate, crude copper azide precipitates immediately. The formation of a 3D-polymeric network, which is built up according to the crystal structure,[S21] leads to the appearance of very fine fibers (observable during electron microscopy, Figure S6) and ultimately to an intergrown polymeric mass of product which is hard to filter and process. This crude product is impure, as it contains basic copper azides. It has to be stored under diluted hydrazoic acid for a certain period of time, leading to the destruction of basic byproducts (Figure S19). That process was monitored by IR spectroscopy, showing a significant reduction of the broad hydroxy band (O–H bond stretching vibration at 3600–3400 cm^{-1}) and sharper remaining bands, e.g. azide band at 2130–2070 cm^{-1} (Figure S18).

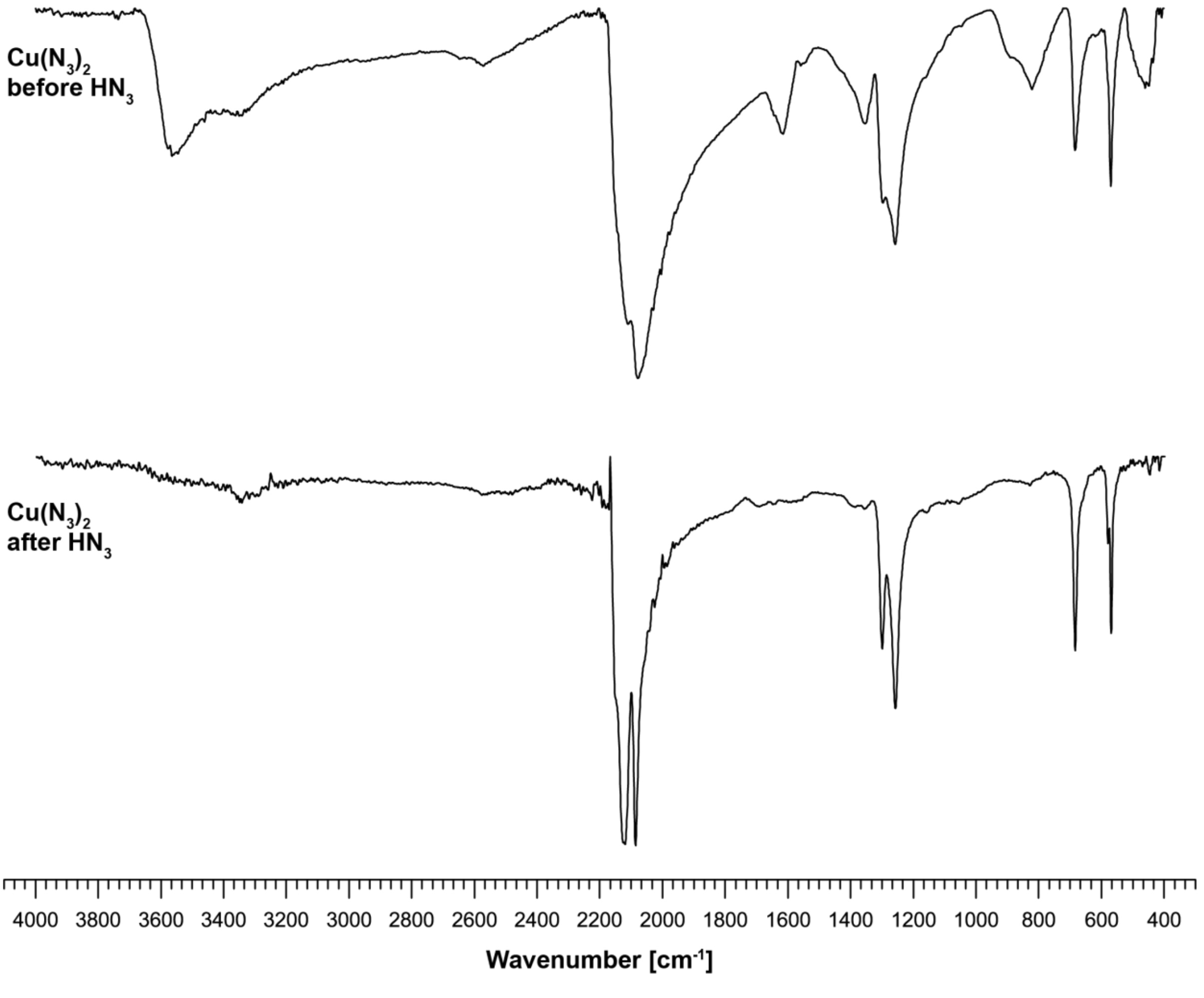

Figure S18. IR spectra of copper(II) azide before and after treatment with diluted HN_3.

$Cu(N_3)_2$ appears to be significantly less sensitive when wet (H_2O, EtOH). It is however very sensitive in the dry state (also when wetted with Et_2O), exploding occasionally during manipulation, e.g. due to slight scratches when being removed from the filter paper (Figure S20).

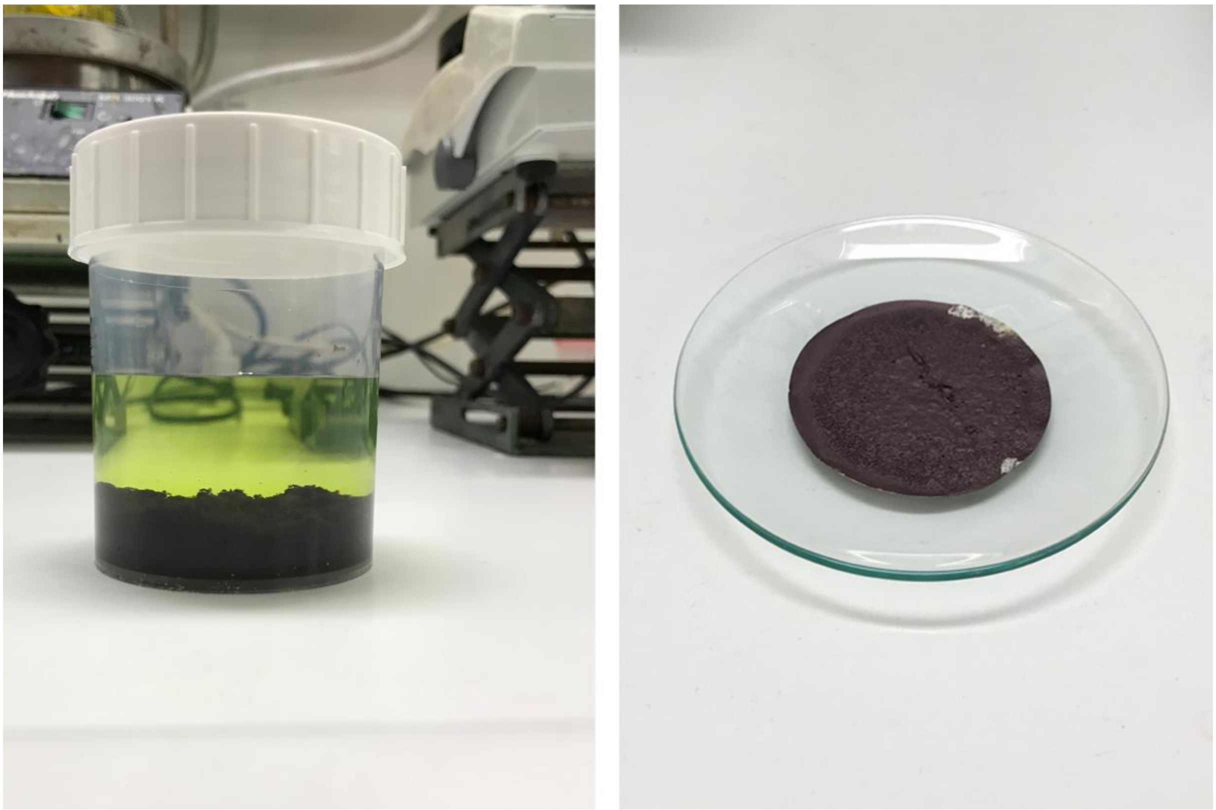

Figure S19. Copper azide under HN_3 (left) and on a filter paper, after washing with ether.

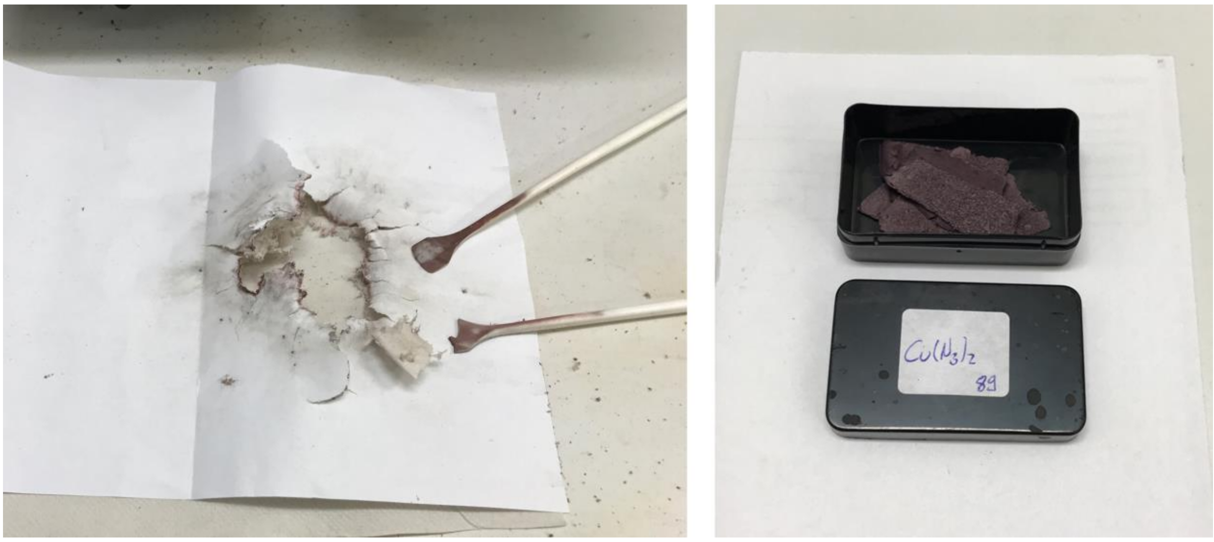

Figure S20. Failed attempt to remove $Cu(N_3)_2$ from a filter paper (left), successful attempt (right).

9. References

[S1] NATO standardization agreement (STANAG) on explosives, impact sensitivity tests, no. 4489, 1st ed., Sept. 17, 1999.

[S2] WIWEB-Standardarbeitsanweisung 4-5.1.02, Ermittlung der Explosionsgefährlichkeit, hier der Schlagempfindlichkeit mit dem Fallhammer, Nov. 8, 2002.

[S3] OZM, http://www.ozm.cz, (accessed January 2020).

[S4] BAM, http://www.bam.de, (accessed January 2020).

[S5] NATO standardization agreement (STANAG) on explosive, friction sensitivity tests, no. 4487, 1st ed., Aug. 22, 2002.

[S6] WIWEB-Standardarbeitsanweisung 4-5.1.03, Ermittlung der Explosionsgefährlichkeit oder der Reibeempfindlichkeit mit dem Reibeapparat, Nov. 8, 2002.

[S7] Impact: insensitive > 40 J, less sensitive ≥ 35 J, sensitive ≥ 4 J, very sensitive ≤ 3 J; Friction: insensitive > 360 N, less sensitive = 360 N, sensitive < 360 N and > 80 N, very sensitive ≤ 80 N, extremely sensitive ≤ 10 N. According to the UN Recommendations on the Transport of Dangerous Goods, (+) indicates not safe for transport.

[S8] M. Straumanis, A. Cīrulis, *Z. Anorg. Allg. Chem.* **1943**, *251*, 315–331.

[S9] J. I. Bryant, H. Rosenwasser, *J. Chem. Educ.* **1962**, *39*, 296.

[S10] T. Costain, F. B. Wells in *Technology of the inorganic azides* (Ed.: H. D. Fair, R. F. Walker), Plenum Press, New York **1977**, pp. 11–54.

[S11] CrysAlisPRO (Version 171.33.41), Oxford Diffraction Ltd., 2009.

[S12] A. Altomare, G. Cascarano, C. Giacovazzo, and A. Guagliardi, *J. Appl. Crystallogr.*, 1992, **26**, 343.

[S13] a) A. Altomare, G. Cascarano, C. Giacovazzo, A. Guagliardi, A. G. G. Moliterni, M. C. Burla, G. Polidori, M. Camalli and R. Spagna, SIR97, 2003; b) A. Altomare, M. C. Burla, M. Camalli, G. L. Cascarano, C. Giacovazzo, A. Guagliardi, A. G. G. Moliterni, G. Polidori and R. Spagna, *J. Appl. Crystallogr.*, 1999, **32**, 115.

[S14] a) G. M. Sheldrick, SHELXL-97, University of Göttingen, Germany, 1997; b) G. M. Sheldrick, *Acta Crystallogr. Sect. A*, 2008, **64**, 112.

[S15] A. L. Spek, PLATON, Utrecht University, The Netherlands, 1999.

[S16] L.J. Farrugia, *J. Appl. Cryst.*, 2012, **45**, 849.

[S17] Empirical absorption correction using spherical harmonics, implemented in SCALE3 ABSPACK scaling algorithm (CrysAlisPro Oxford Diffraction Ltd., Version 171.33.41, 2009).

[S18] APEX3, Bruker AXS Inc., Madison, Wisconsin, USA.

[S19] J. R. Rodriguez-Carvajal, Abstracts of the Satellite Meeting on Powder Diffraction of XV Congress of the IUCr, Toulouse, France, 1990, 127.

[S20] C. M. Csernica in *40th Int. Pyro. Symp.*, Colorado **2014**, pp. 114–133.

[S21] I. Agrell, *Acta Chem. Scand.* **1967**, *21*, 2647–2658.
